# Knockdown of Parkinson’s disease-related gene ATP13A2 reduces tumorigenesis via blocking autophagic flux in colon cancer

**DOI:** 10.1186/s13578-020-00506-z

**Published:** 2020-12-11

**Authors:** Qian Chen, Li Zhong, Chao Zhou, Yan Feng, Quan-xing Liu, Dong Zhou, Xiao Lu, Guang-Sheng Du, Dan Jian, Hao Luo, Dong Wang, Hong Zheng, Yuan Qiu

**Affiliations:** 1 Cancer Center of Daping Hospital, Third Military Medical University (Army Medical University), Chongqing, 400037 China; 2 Department of General Surgery, Xinqiao Hospital, Third Military Medical University (Army Medical University), Chongqing, 400037 China; 3 Department of Thoracic Surgery, Xinqiao Hospital, Third Military Medical University (Army Medical University), Chongqing, 400037 China; 4 State Key Laboratory of Trauma, Burn and Combined Injury, Third Military Medical University (Army Medical University), Chongqing, 400037 China

**Keywords:** ATP13A2, Colon cancer, Autophagy, Tumorigenesis

## Abstract

**Background:**

Accumulating evidence shows that Parkinson’s disease is negatively associated with colon cancer risk, indicating that Parkinson’s disease family proteins may be involved in the initiation of colon cancer. Here, we aimed to identify a Parkinson’s disease-related gene involved in colon cancer, elucidate the underlying mechanisms, and test whether it can be used as a target for cancer therapy.

**Methods:**

We first screened colon cancer and normal tissues for differential expression of Parkinson’s disease-associated genes and identified *ATP13A2*, which encodes cation-transporting ATPase 13A2, as a putative marker for colon cancer. We next correlated ATP13A2 expression with colon cancer prognosis. We performed a series of ATP13A2 knockdown and overexpression studies in vitro to identify the contribution of ATP13A2 in the stemness and invasive capacity of colon cancer cells. Additionally, autophagy flux assay were determined to explore the mechanism of ATP13A2 induced stemness. Finally, we knocked down ATP13A2 in mice using siRNA to determine whether it can be used as target for colon cancer treatment.

**Results:**

Colon cancer patients with high ATP13A2 expression exhibit shorter overall survival than those with low ATP13A2. Functionally, ATP13A2 acts as a novel stimulator of stem-like traits. Furthermore, knockdown of ATP13A2 in HCT116 resulted in decreased levels of cellular autophagy. Additionally, bafilomycin A1, an autophagy inhibitor, reversed the ATP13A2-induced stemness of colon cancer cells. Lastly treatment with ATP13A2 siRNA reduced the volume of colon cancer xenografts in mice.

**Conclusions:**

The PD-associated gene *ATP13A2* is involved in colon cancer stemness through regulation of autophagy. Furthermore, ATP13A2 is a novel prognostic biomarker for colon cancer and is a potential target for colon cancer therapy.

## Background

Colon cancer is one of the most prevalent cancers worldwide and causes 551,269 deaths each year [1] Accumulating evidence supports the hypothesis that colorectal cancer stem cells (CSC), which exhibit stemness (i.e., self-renewal and pluripotency), have the ability to initiate and sustain tumor growth, metastasis, and resistance to therapy [2]. However, CSC can be derived from non-CSC that have undergone genetic modification [3]. When faced with endogenous or exogenous stress, tumor cells maintain their stemness and self-renew through autophagic degradation of misfolded proteins and aging organelles [4, 5] Therefore, identifying novel factors that target autophagy-mediated stemness of colon cancer cells has been proposed as a therapeutic strategy for colon cancer.

Parkinson’s disease is characterized by apoptosis of dopamine producing cells in the substantia nigra, which is negatively correlated with the incidence of colon cancer risk [6, 7]. However, Parkinson’s disease and colon cancer share several other risk factors including cellular aging, DNA damage in response to oxidative stress, and metabolic dysregulation [8]. Recent genome-wide association studies have revealed the correlation of more than a dozen loci (including PARK1/4, PARK2, PARK5, PARK6, PARK7, PARK8, ATP13A2, PARK15, and GBA) with familial Parkinson’s disease [9, 10]. Given the inverse correlation between Parkinson’s disease and colon cancer risks, some of these genes may be involved in the initiation of colon cancer. Our previous research suggested that Parkinson’s disease family proteins, such as PARK15 and PARK7, are associated with tumorigenesis of non-small cell lung cancer [11], However, the Parkinson’s disease-associated proteins that are involved in the initiation of colon cancer have not yet been determined.

Therefore, in this study, we first aimed to find a Parkinson’s disease family protein that is differentially expressed in colon cancer and normal tissue, and we were able to identify cation-transporting ATPase 13A2 (ATP13A2) as a putative biomarker for colon cancer. We next aimed to correlate ATP13A2 with colon cancer patient prognosis to determine whether it can be used as a prognostic predictor. We also aimed to understand the role of ATP13A2 in maintaining the colon cancer invasive capacity and stemness. Finally, we tested whether ATP13A2 can be targeted in colon cancer therapy in mice. The findings of this study illustrate the role of ATP13A2 in colon cancer and show that ATP13A2 is a putative novel prognostic marker as well as a novel potential therapeutic target in colon cancer.

## Material and methods

### Patients and tissue specimens

Twenty-seven pairs of tumor and corresponding normal tissue were obtained from patients with colon cancer who underwent surgical resection at Xinqiao Hospital Army Medical University from January to December 2016. Two tissue microarrays consisting of 99 colon cancer tissues that were not exposed to radiotherapy or chemotherapy prior to surgery were purchased from BioChip (Shanghai, China). All patients were provided with informed consent and followed up five years. The clinical information was shown in Table 1. An independent cohort of 25 tumor and 32 normal tissues from the ONCOMINE database was downloaded from https://www.oncomine.org/resource/login.html. An independent cohort of 275 tumor and 349 normal tissues from GEPIA database was downloaded from http://gepia.cancer-pku.cn/index.html. The oncomine and GEPIA data were collected from GEO data base (GSE8671) and TCGA database.

**Table 1:** The clinical features of the colon cancer specimens used in this study

Feature	WHO grade		
	I (n = 7)	II (n = 43)	III (n = 49)
Gender			
Male	6	21	26
Female	1	22	23
Age at diagnosis			
< 60	1	4	13
≥ 60	6	39	36
Invasive depth			
Submucosa	1	0	0
Muscular layer	1	3	2
Serous layer	4	34	40
Whole layer	1	6	7
Metastasis			
Yes	0	0	3
No	7	43	46
Lymph node metastasis			
Yes	1	15	22
No	6	28	27
Location			
*Left colon*	4	17	23
Right colon	3	26	26

### Cells and reagents

Human colorectal cancer cell lines, SW480 (CVCL-0546) and HCT-116 (ATCC CCL-247), normal colon epithelial cell line CCD 841CoN (ATCC CRL-1790) were obtained from the American Type Culture Collection (ATCC) and maintained in a humidified atmosphere of 5% CO2 at 37 °C in the DMEM suggested by ATCC supplemented with fetal bovine serum (FBS) (Gibco, Invitrogen, Carslbad, CA) and penicillin and streptomycin. bafilomycin A1 was purchased from Selleckchem (S1413; Houston, TX). DOTAP Liposomal Transfection Reagent was purchased from Sigma (11,202,375,001; Germany).

### Overexpression and stable knockdown of ATP13A2 in cancer cells

For the establishment of a cell line that stably overexpresses ATP13A2, an LV5 (EF-1a/GFP/Puro/Amp) lentiviral vector containing the human ATP13A2 was used to transfect cancer cells. Lentiviral particles packaged with an empty vector served as the negative control. To generate the stable ATP13A2 knockdown cells, one pairs of self-complementary hairpin DNA fragments targeting ATP13A2 mRNA and control DNA were synthesized and cloned into an LV-3 (pGLVH1/GFP/Puro) lentiviral vector. The ATP13A2-targeting siRNA sequence was si*ATP13A2*: 5′-GCCUCUGAACGAUAUUGTAAT-3′; and the non-targeting scrambled sequence was siNC5′-UUCUCCUAACUTUTCACUTTT-3′. Fresh culture medium containing 4 μg/mL puromycin was added to select stable puromycin-resistant cells.

### Western blotting, reverse transcription, and qPCR

Western blotting, reverse transcription, and qPCR were carried out as previously described. The primary antibodies used for western blotting were: rabbit anti-human ATP13A2 (1:500); rabbit anti-human organic cation/carnitine transporter 4 (OCT4; 1:1000); rabbit anti-human transcription factor SOX-2 (SOX2; 1:1000); anti-sequestosome-1 (SQSTM1; 1:1000; 232,145); anti-autophagy protein 5 (ATG5; 1:1000, 12994 T); rabbit anti-Beclin-1 (1:1000, 3495 T); rabbit anti-human glyceraldehyde-3-phosphate dehydrogenase (GAPDH; 1:1000) from Cell Signaling Technology (Danvers, MA); and anti-human microtubule-associated protein 1A/1B-light chain 3 (LC3; 1:100, PA1-16,930) from Thermo Fisher Scientific (Waltham, MA). The sequences of primers used in this study are shown in Additional file 1: Table S1.

### Self-renewal and colony formation assay

Single cells were harvested and seeded into 96-well plates (40 cells/well). On day 7, the formation of spheres was examined under the microscope, and the efficiency of sphere formation per well determined by counting the number of spheres that consisted of at least 30 cells. For colony formation assay, cells (400 cells/well) were seeded into 6-well plates in 2 mL complete medium (DMEM supplemented with 10% FBS). The plates were further incubated for 10 days at 37 °C with 5% CO2 until colonies were visible. The colonies were stained with 0.01% crystal violet and counted under an inverted microscopy.

### Immunohistochemistry (IHC) and scoring

IHC detection of ATP13A2, SQSTM1 and LC3B were performed using the Dako Envision FLEX + system (Dako, Berlin, Germany). Paraffin sections were deparaffinized. Antigen retrieval was performed by heating the samples in citrate buffer (pH 6.0) in the microwave for 15 min, then returning the samples to room temperature, followed by washing with phosphate-buffered saline (PBS). The samples were blocked with the Dako REAL Peroxidase-Blocking Solution for 15 min. The slides were incubated at 4 °C overnight with the ATP13A2 antibody (1:100; orb158100, Biorbyt, Cambridge, UK), the SQSTM1 antibody (1:1000; 88588, CST, Danvers, MA) or the LC3B antibody (1:400; ab221794, Abcam, Cambridge, UK), followed by incubation (30 min) with the secondary antibody. Slides were stained with 3,3′-diaminobenzidine tetrahydrochloride (DAB) for 2 min. The percentages of cells that were positive for the markers were scored as follows: 0‒5%, no positive cells; 1, < 25% positive cells; 2, 25‒50% positive cells; 3, 50‒75% positive cells; 4, 75‒100% positive cells. The staining intensity was scored as follows: 0, no positive cells; 1, weak staining; 2, moderate staining; and 3, strong staining. The immunohistochemical staining score was obtained by multiplying the percentage score by the intensity (0, 1, 2, 3, 4, 6, 8, 9, or 12). Scores were analyzed using the statistical software X-tile with a score of 8 as the cut-off value [12].

### Tumor implantation

SW480 cells transfected with the pLVX-eGFP-linker-luciferase lentivirus (1 × 10^6^ cells) suspended in 100 μL PBS were subcutaneously injected into the groin surfaces of anesthetized 5-week-old female nude mice (n = 3). Ten days after implantation, the developed tumors were treated with control siRNA (siNC) or ATP13A2 siRNA (si*ATP13A2*) together with DOTAP Liposomal Transfection Reagent (50 μg siRNA + 25 μg DOTAP). The siRNA-DOTAP solution (200 μL) was directly injected around the base of each tumor as previously described [13]. Ten days after siRNA injection, the growing xenograft tumors were detected and measured by bioluminescence imaging using an In Vivo Image System (IVIS) Spectrum (PerkinElmer, Waltham, MA). Twenty days after treatment, all the animals were sacrificed, and subcutaneous xenografts were used for histological examination. The animal experiments were approved by the Institutional Animal Care and Use Committee of Xinqiao Hospital, Third Military Medical University in accordance with the Guide for the Care and Use of Laboratory Animals.

### Autophagy flux assay

HCT-116 cells in 6-well plates were transfected with 1 × 10^5^/well EGFP-LC3 adenovirus (HANBIO, Shanghai, China). After culture on scaffolds for 24 h, the accumulation and distribution of EGFP-LC3 puncta in HCT-116 cells were observed by confocal microscopy. LSM 780 NLO microscope systems and the ZEN Imaging Software (Zeiss, Oberkochen, Germany) were used for image acquisition and export.

### Statistical analysis

Statistical analyses were performed using the SPSS software 19.0. The normality of distribution was estimated through the Kolmogorov–Smirnov test. The correlation of ATP13A2 and clinicopathological features of patients were assessed by the Pearson χ^2^ test. Survival curves were obtained by the Kaplan–Meier method, and comparisons were made by the log-rank test. Paired Student’s *t*-test for two groups and one-way ANOVA for multiple group data were applied in this study. Statistical difference was considered significant if the *P* value was less than 0.05 (assigned as *), less than 0.01 (assigned as **), or less than 0.001 (assigned as ***). All experiments were carried out at least three times with triplicate samples.

## Results

### ATP13A2 is highly expressed in human colon cancer tissues

**Fig. 1:**
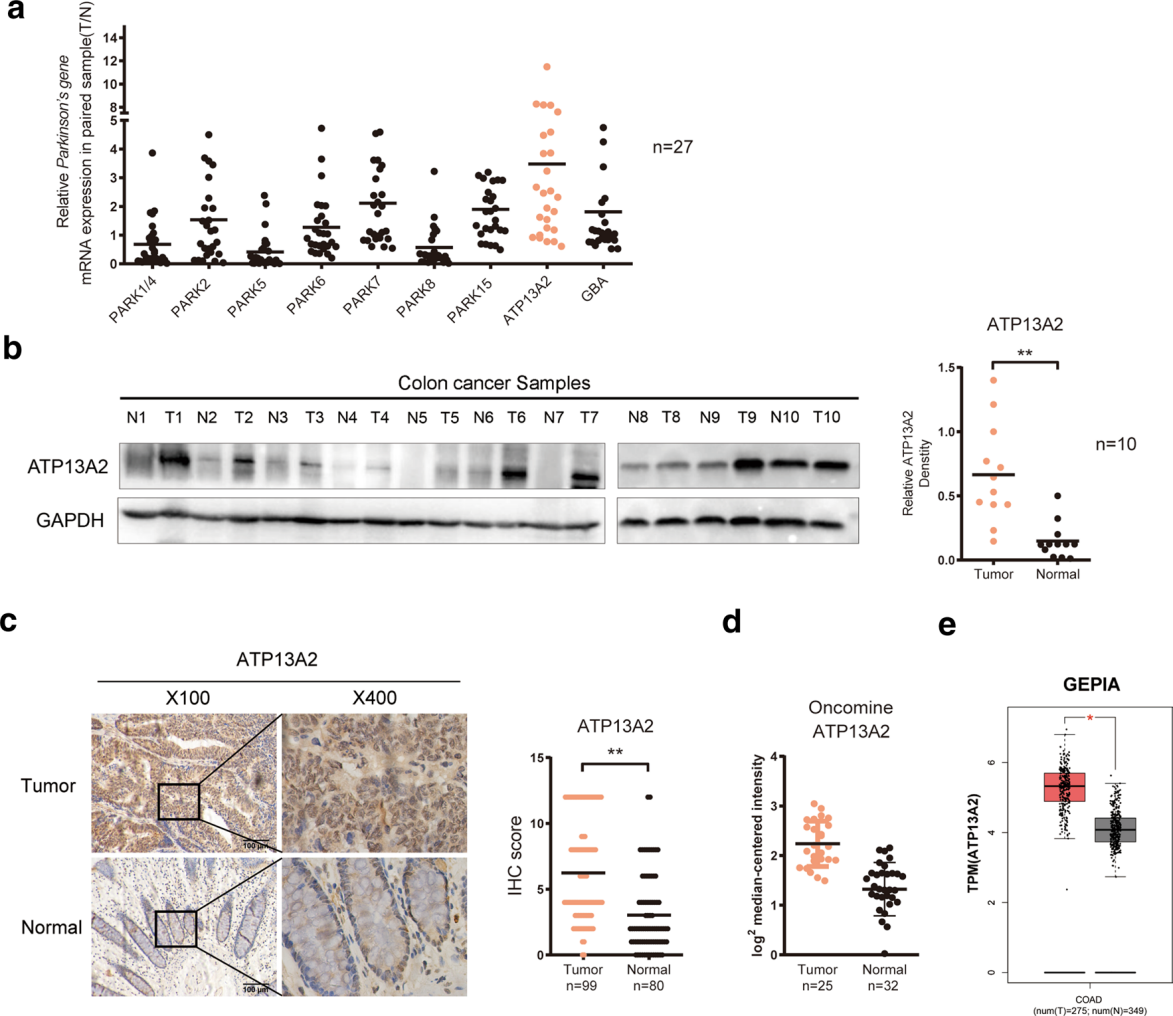
ATP13A2 was highly expressed in colon cancer tissue samples. **a** Relative expression (Tumor/Normal) of Parkinson’s disease-associated genes (*PARK1/4*, *PARK2*, *PARK5*, *PARK6*, *PARK7*, *PARK8*, *PARK15*, *ATP13A2*, *GBA*) in 27 paired colon cancer specimens. **b** Western blotting (*Left panel*) and qualification (*Right panel)* analysis show ATP13A2 expression levels in 10 colon cancer specimens. **c** Representative images of ATP13A2 immunohistochemical staining (brown color) in colon cancer samples (T) and normal colon tissue (N) (scale bar = 100 μm). **d–e** The relative expression of ATP13A2 in CRC cancer and normal tissue from the ONCOMINE and GEPIA databases. *P < 0.05; **P < 0.01

To investigate the role of Parkinson’s disease-associated proteins in colon cancer progression, the transcript levels of genes that encode these proteins, including *PARK1/4*, *PARK2*, *PARK5*, *PARK6*, *PARK7*, *PARK8*, *PARK15*, *ATP13A2* and *GBA*, were evaluated by qPCR. We found that the mRNA levels of *ATP13A2* and *PARK7* were significantly higher in colon cancer tissue than in normal tissue (Fig. 1a). In our previous study, we have revealed that PARK7 is associated with colon cancer invasion and progression [14, 15], but the role of ATP13A2 in colon cancer initiation and progression remains unknown.

Western blotting also showed that the expression of ATP13A2 was significantly higher in tumor tissues than that in the paired normal tissues (Fig. 1b). IHC using a 99-case colon cancer cohort (Cohort-99) revealed that in 80 paired cases, the staining score of ATP13A2 was remarkably higher in the tumor tissue than in the corresponding adjacent non-cancerous tissue (Fig. 1c). In addition, ATP13A2 mRNA expression from the ONCOMINE and GEPIA databases was noted to be higher in colon cancer tissues than in the normal tissues (Fig. 1d, e). Furthermore, ATP13A2 was also detected higher in CRC cell lines than normal colon epithelial cell line (CCD 841CoN) (Additional file 1: Figure S1). Taken together, these data indicated that ATP13A2 expression was significantly higher in colon cancer tissues than in adjacent colon tissues.

### High ATP13A2 expression in colon cancer specimens is associated with poor patient outcome

**Fig. 2:**
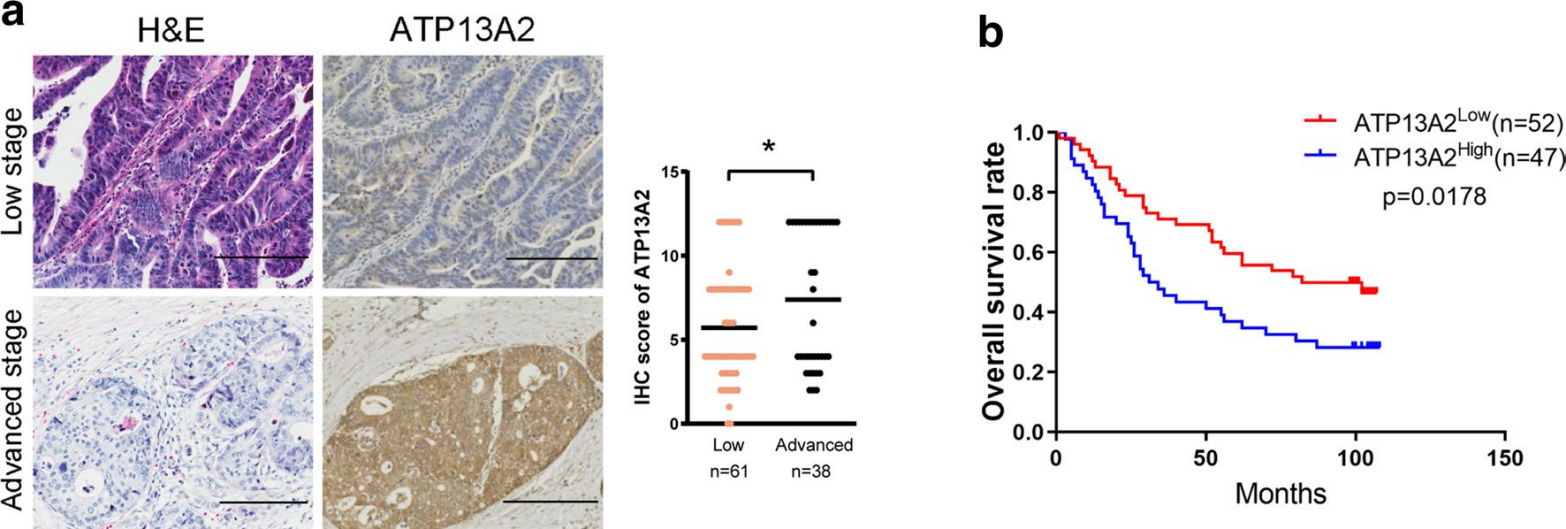
ATP13A2 expression and its prognostic value in colon cancer. **a** Representative images and scores of ATP13A2 staining in tumors and in normal colon tissue of paired colon cancer specimens from patients with low and advanced TNM stages (scale bar = 100 μm). Low stage indicated Stage I and II, while high stage indicated Stage III and IV. **b** Overall survival rates of colon cancer patients with low and high ATP13A2 expression. *, *P* < 0.05

We next analyzed the association of ATP13A2 expression with the lifespan and tumor-node-metastasis (TNM) stage of colon cancer patients using the clinicopathological features of the patients (Table 1). The results showed that ATP13A2 expression levels were higher in advanced stages than those in early stages of colon cancer (Fig. 2a). More importantly, the level of ATP13A2 expression in colon cancer was positively correlated with lymph node metastasis (P = 0.019, Table 2). Kaplan–Meier survival curves showed that the overall survival of patients with high ATP13A2 expression (ATP13A2^high^) was significantly shorter than that of patients with low ATP13A2 expression (ATP13A2^low^) tumors (P = 0.0178, Fig. 2b). The 5 year-survival rate of patients with high ATP13A2 was 37%, while those with low ATP13A2 was 60%. Furthermore, univariate and multivariate analyses revealed that the expression of ATP13A2 is an independent indicator for the overall survival rate of patients with colon cancer (Table 3). Therefore, ATP13A2 expression in colon cancer appears to serve as a predictor for prognosis of colon cancer patients.

**Table 2:** The correlation between ATP13A2 expression and clinicopathological factors of patients with colon cancer was used in this study

Feature	ATP13A2		P
	Low (n = 52)	High (n = 47)	
Gender			P = 0.948
Male	28	25	
Female	24	22	
Age at diagnosis			P=0.992
< 60	10	9	
≥ 60	42	38	
Invasive depth			P = 0.451
Submucosa	1	0	
Muscular layer	4	2	
Serous layer	42	36	
Whole layer	5	9	
Location			P = 0.287
Left	31	23	
Right	21	24	
T stage			P = 0.174
T_1–3_	47	38	
T_4_	5	9	
N stage			P = 0.019
N0	38	23	
N1-2	14	24	
M stage			P = 0.618
M0	50	46	
M1	2	1	
Histological grade			P = 0.196
Well	3	4	
Moderate	22	21	
Poor	27	22

**Table 3:** Univariate and Multivariate analysis for overall survival in colon cancer

Factors	Univariate		Multivariate	
	HR (95% CI)	*P* value	HR (95% CI)	*P* value
Gender	1.117 (0.672–1.856)	0.669	0.899 (0.511–1.582)	0.712
Age	1.019 (0.994–1.044)	0.315	1.016 (0.992–1.040)	0.195
Location	0.610 (0.360–1.031)	0.065	0.549 (0.319–0.943)	0.030
ATP13A2 expression	1.745 (1.048–2.907)	0.032	2.272 (1.319–3.915)	0.003
Grade	1.452 (0.942–2.230)	0.088	1.664 (1.053–2.631)	0.029
TNM Stage	2.612 (1.565–4.360)	0.000	2.777 (1.587–4.860)	0.000

### ATP13A2 is closely related to the stemness and invasive capacity of colon cancer cells

**Fig. 3:**
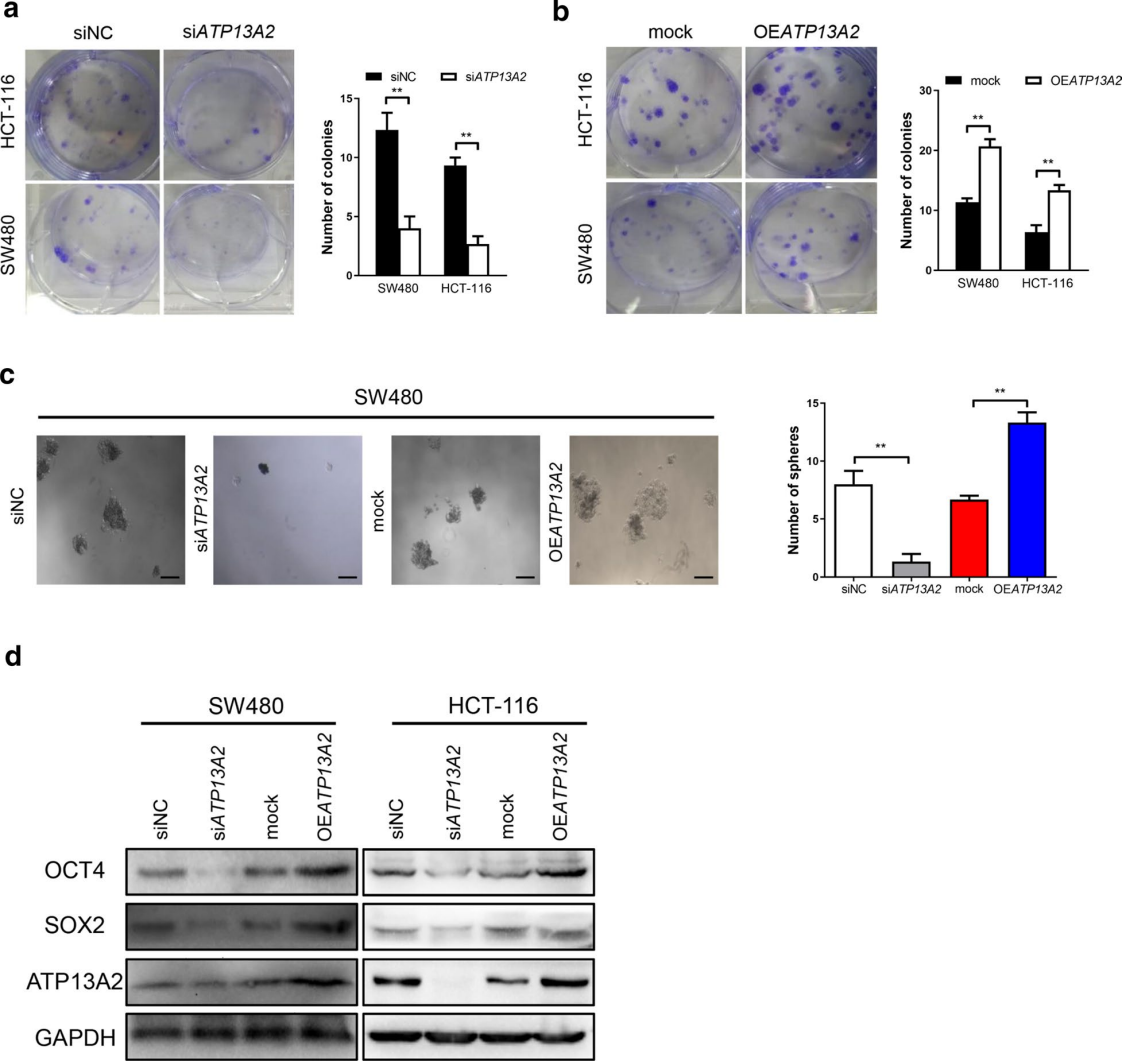
ATP13A2 promotes stem-like traits of colon cancer cells in vitro. **a** Representative images (*Left panel*) and colony counts (*Right panel*) of two colon cancer cell lines (HCT-116, SW480) treated with si*ATP13A2* and siNC. **b** Representative images (*Left panel*) and colony counts (*Right panel*) in two colon cancer cell lines (HCT-116, SW480) transfected with the ATP13A2 overexpression vector (OE*ATP13A2*) and the empty vector (mock). **c** Representative images (*Left panel*) and sphere counts (*Right panel*) of si*ATP13A2*-SW480 and siNC-SW480, OE*ATP13A2*-SW480 and mock-SW480 cell. Scale bar = 100 µm. **d** Western blotting for SOX2 and OCT4 in SW480 and HCT-116 cells with down- and upregulated-ATP13A2 respectively, compared with control cells. **, P < 0.01

Studies have shown that stemness of tumor cells promotes colon cancer initiation and progression [16]. Therefore, we explored the potential relationship between ATP13A2 expression and stemness of colon cancers through knockdown and overexpression studies. We found that that the number of colonies formed by the si*ATP13A2*-colon cancer cells significantly decreased relative to the number of colonies formed by the siNC-colon cancer cells (Fig. 3a). Meanwhile, overexpression of ATP13A2 increased the number of colonies formed by the colon cancer cells compared to those formed by the mock cells (Fig. 3b). Furthermore, the volume and number of tumor spheres derived from the si*ATP13A2*-colon cancer cells were smaller than those derived from the siNC-colon cancer cells, and overexpression of ATP13A2 had reverse effects (Fig. 3c). Additionally, western blotting analysis revealed that overexpression of ATP13A2 increased the levels of CSC markers including SOX2 and OCT4, while knockdown of ATP13A2 reduced the levels of these markers (Fig. 3d).

**Fig. 4:**
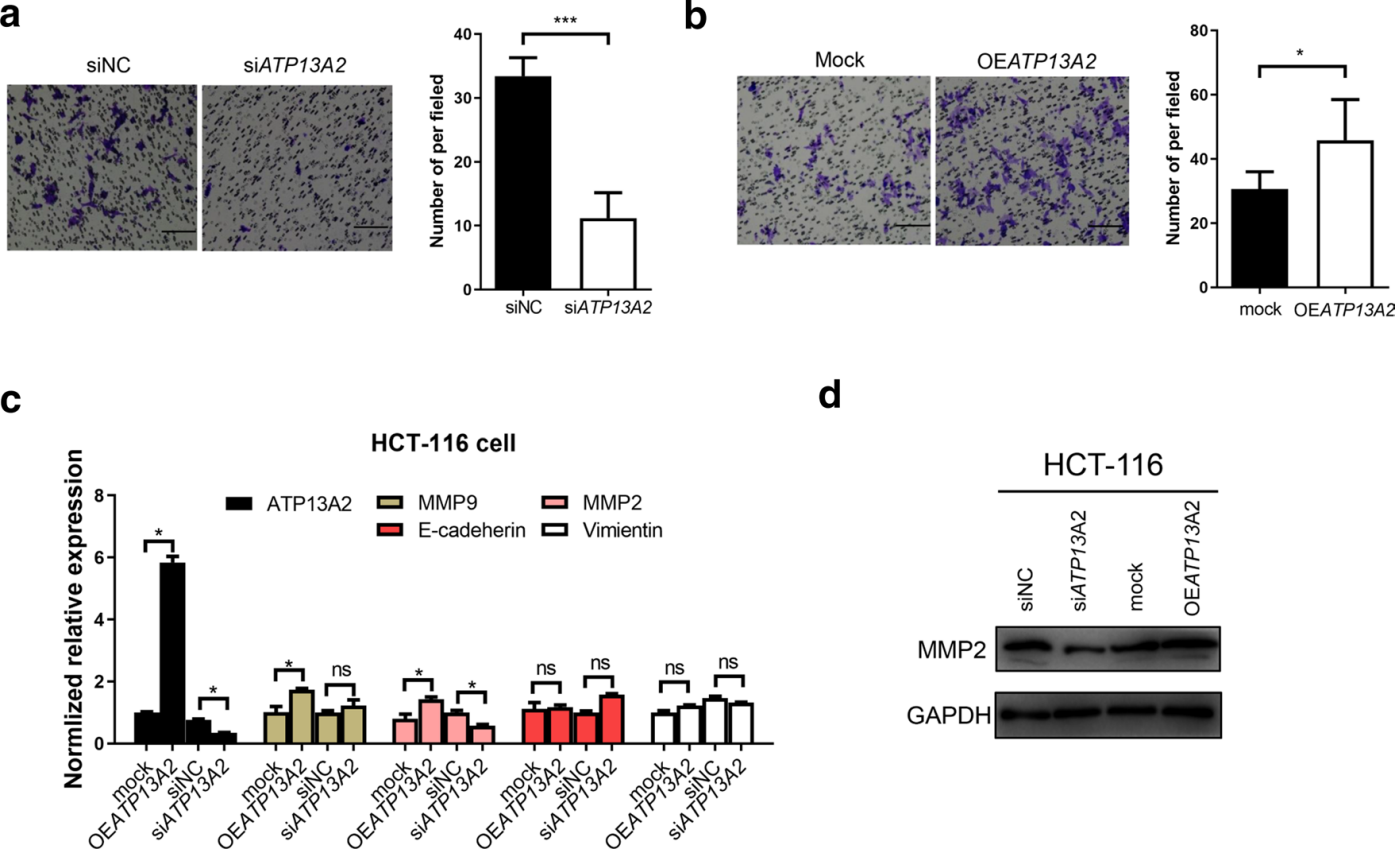
ATP13A2 increases the invasive capacity of colon cancer cells in vitro. **a** Representative images (*Left panel*) and number of invasive cells (Right panel) of HCT-116 cell treated with siNC or si*ATP13A2*. **b** Representative images (Left panel) and number of invasive cells (Right panel) in cells that overexpress ATP13A2 as and the control cells (ctrl). **c** The relative expression of ATP13A2, MMP2, MMP9, E-cadherin, Vimentin in mock- and OE*ATP13A2*; siNC and si*ATP13A2*- HCT-116 cell. **d** The relative protein of MMP2 in mock- and OE*ATP13A2*; siNC and si*ATP13A2*- HCT-116 cell. *, P < 0.05; ***, P < 0.001. ns, no significant

Moreover, Table 2 shows that high expression level of ATP13A2 in colon cancer tissues was linked to high lymph node metastasis rates. We next investigated the relationship between ATP13A2 expression and the invasive capacity of colon cancer cells. Expectedly, the Transwell assay showed that decreased ATP13A2 level significantly impaired the mobility of colon cancer cells (Fig. 4a), while elevated ATP13A2 levels markedly enhanced their mobility (Fig. 4b). EMT and MMPs are two major pathways which is correlated with invasion. Therefore, we examined MMP related gene (MMP2, MMP9) and EMT (E-cadherin, Vimeitin) related gene, we found MMP2 was significantly increased in OE*ATP13A2* than that in mock cell, while decreased MMP2 in si*ATP13A2* (Fig. 4c and d). These data show that ATP13A2 is crucial to the initiation and progression of colon cancer.

### ATP13A2 knockdown decreased stemness by blocking autophagy in colon cancer cells

**Fig. 5:**
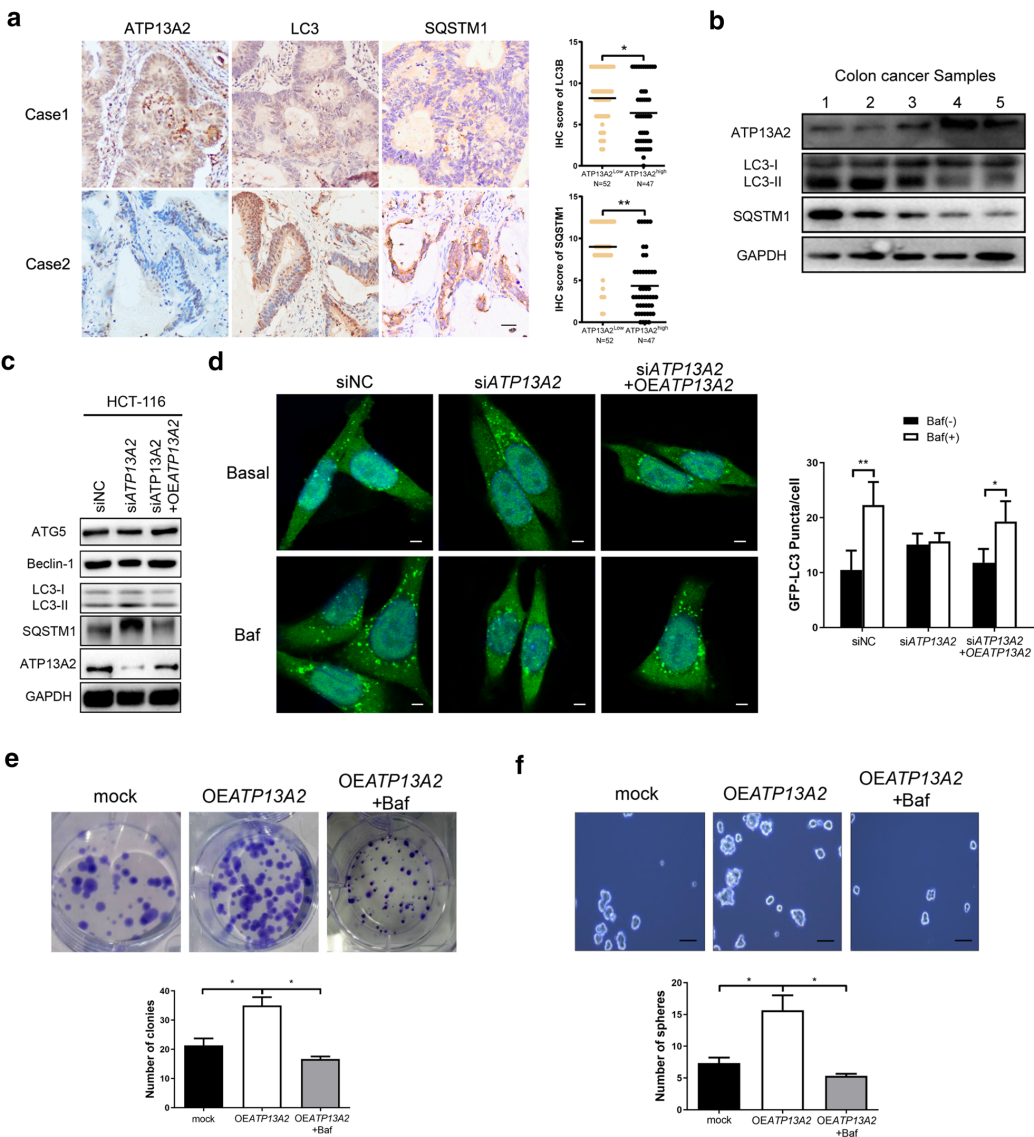
Involvement of ATP13A2 in the stemness of colon cancer cells through regulation of autophagy. **a** Representative immunohistochemical staining of ATP13A2, LC3, SQSTM1 (*Left panel*) and statistical analysis (*Right panel*) in colon cancer tissues (n = 99). Scale bar = 50 µm. (**b**) ATP13A2, LC3 and SQSTM1 were detected by western blotting (WB) in five fresh human colon cancer samples. **c** The protein expression of ATP13A2, SQSTM1, LC3, Beclin-1, and ATG5 in ATP13A2-knockdown HCT-116 cell groups, in ATP13A2-knockdown groups rescued by ATP13A2 overexpression, and in control cells. **d** The representative image (*Left panel*) and number (*Right panel*) of green puncta (LC3 protein) from HCT-116-siNC and HCT-116-si*AT*P13A2 treated with and without bafilomycin; HCT-116 cells were co-transfected with indicated siRNA and GFP-LC3 plasmid. Some si*ATP13A2* cultures were rescued by the overexpression of ATP13A2 (OE*ATP13A2)*. After 48 h, cultures were treated with dimethyl sulfoxide (DMSO) or bafilomycin (5 nM) for 2 h. Scale bar = 5 μm. N = 5, with 5–10 cells imaged per experiment, values are means ± SD. **e–f** The number of colonies (**e**) and spheres (**f**) in the mock group, OE*ATP13A2* group, and OE*ATP13A2* + bafilomycin (5 nM) group. *P < 0.05; **P < 0.01;

Autophagy is a lysosome-mediated process that plays a complex role in sustaining tumor stemness [17]. Furthermore, the localization of ATP13A2 within the lysosomal membrane suggests a potential role for ATP13A2 in the modulation of autophagy. Mutation or knockdown of ATP13A2 can cause lysosomal dysfunction, including reduced lysosomal acidification and impaired autophagic flux, indicating that ATP13A2 is closely related with autophagy [18]. Therefore, it is worthwhile to explore the expression of ATP13A2 and autophagy in colon cancer cells. IHC staining was performed on the 99-case colon cancer cohort to explore the relationship between the expression levels of ATP13A2, LC3 and SQSTM1, the marker of autophagy. We found that the expression of was higher the autophagy marker (LC3 and SQSTM1) in ATP13A2 low colon cancer cells, but lower in ATP13A2 high colon cancer cells (Fig. 5a). Additionally, western blotting analysis showed that these two markers of autophagic flux, was inversely related with ATP13A2 expression in colon cancer samples (Fig. 5b), suggesting that ATP13A2 is involved in regulating autophagy in colon cancer cells. Moreover, knockdown of ATP13A2 in HCT-116 cells led to the accumulation of LC3-II and SQSTM1/p62, and this effect can be rescued by overexpression of ATP13A2. At the same time, no significant changes were observed in the expression of ATG5 and Beclin-1 (Fig. 5c).

To further investigate the role of ATP13A2 in autophagy regulation of colon cancer cells, HCT-116 cells were infected with LC3-GFP adenovirus. The use of 5 nM bafilomycin A1 results in the optimal neutralization of lysosomal pH [19], without eliciting signs of stress or injury. After 1 h in the presence of bafilomycin A1, HCT-116 cells that were treated with siNC showed a significant increase in the number of LC3-GFP puncta. However, HCT-116 cells treated with ATP13A2 targeting siRNA showed no increase in the number LC3-GFP puncta after bafilomycin A1 treatment; this was rescued by the overexpression of ATP13A2 in colon cancer cells, indicating that ATP13A2 knockdown decreased basal autophagic flux (Fig. 5d). Furthermore, overexpression of ATP13A2 significantly increased the number and size of colonies and spheres of HCT-116 cells, and this effect was reversed by bafilomycin (Fig. 5e, f). Taken together, these data suggest that ATP13A2 knockdown blocks autophagic flux, resulting in reduced stemness of colon cancer cells. Therefore, these results indicate that ATP13A2 is involved in the regulation of stemness in colon cancer cells by regulating autophagy.”

### Inhibition of xenograft tumor proliferation by in vivo treatment with ATP13A2 siRNA

**Fig. 6:**
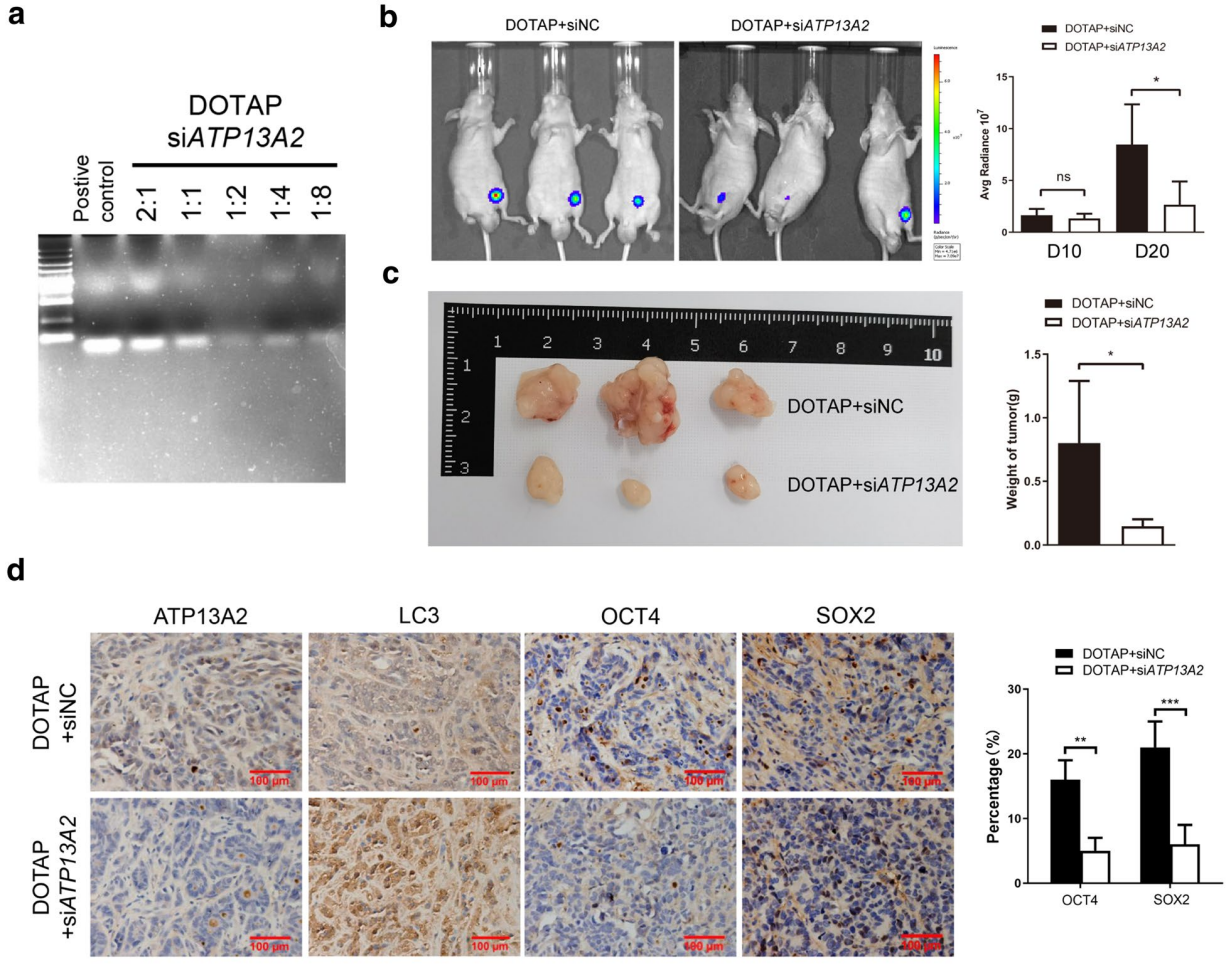
Inhibition of xenograft tumor proliferation by in vivo treatment with *ATP13A2* siRNA. **a** Gel retardation assay shows the effects of using different ratios of DOTAP and si*ATP13A2*. **b** Nude mice (n = 3) were implanted with SW480 cells, and after 10 days, the mice were treated with siNC or si*ATP13A2* mixed with DOTAP. Bioluminescence (*Left panel*) and qualification (*Right panel*) images of tumors in these mice at 20 day after incubation were shown. **c** Representative image and quantitative analysis of weight of the xenograft tumor were shown when mice were sacrificed. **d** Representative images (*Left panel*) and quantification (*Right panel*) of SOX2, OCT4, LC3, and ATP13A2 in xenograft tumors treated with siNC or si*ATP13A2* mixed with DOTAP (n = 3). *, *P* < 0.05; **, *P* < 0.01; ***, *P* < 0.01; ns, no significant

To examine whether ATP13A2 is a possible potential target for cancer therapy, colon cancer cells were transfected with luciferase and implanted into nude mice (n = 3), and the tumors were allowed to develop for 10 days. We used DOTAP (1,2-dioleoyl-3-trimethylammonium-propane), a cationic liposome, for stable delivery of si*ATP13A2* to cancer cells. Gel retardation assay shows that the optimum mass ratio of DOTAP to siRNA was 1:2 (Fig. 6a). Next, siNC (control) or si*ATP13A2* was mixed with DOTAP, and the mixture was injected once around the base of each xenograft tumor. Twenty days after tumor implantation, bioluminescent imaging detected smaller tumors in mice treated with si*ATP13A2* than in mice treated with siNC (Fig. 6b) and the xenograft tumors treated with DOTAP + si*ATP13A2* had a slower growth as compared to those treated with DOTAP + siNC (Fig. 6c). Immunohistochemical analyses also showed that the numbers of ATP13A2-, LC3-, and cancer stem cell-positive cells were lower in the si*ATP13A2*-injected tumors (Fig. 6d), indicating that ATP13A2 was involved in sustaining the stemness of colon cancer via autophagy in vivo. Therefore, silencing of ATP13A2 in vivo significantly reduced tumor sizes, suggesting that ATP13A2 is a potential target for cancer therapy.

## Discussion

Accumulating evidence suggest that the stemness of cancer cells is responsible for tumor initiation, progression, and drug-resistance [20]. In this study, to the best of our knowledge, we report for the first time that ATP13A2 functions in the promotion of colon cancer stemness and is a predictive factor of colon cancer patient outcomes. Furthermore, we show that ATP13A2 can be considered as a therapeutic target in colon cancer. si*ATP13A2* in colon cancer cells also significantly reduced the CSC subpopulation and stemness-associated molecules. Further investigation showed that ATP13A2 regulates the stemness of colon cancer cells by regulating autophagy.

Recent studies have shown that the past medical history of Parkinson’s disease more than 1 year before the index-date of colon cancer was associated with lower risks of acquiring colon cancer, suggesting an inverse correlation between the incidence of Parkinson’s disease and of colon cancer [21] ATP13A2 mutation leads to α-synuclein accumulation, which is associated with gut microbiota formation and a higher risk of Parkinson’s disease [6, 22, 23]. Interestingly, the gut microbiota might also serve as a common pathway in the pathogenesis of both Parkinson’s disease and colon cancer [24]. Herein, we found that ATP13A2 was highly expressed in colon cancer tissues than in paired normal tissues, and that higher ATP13A2 expression in colon cancer cells was positively related with the stemness and poor prognosis of colon cancer, indicating that high expression of ATP13A2 is associated with higher colon cancer risks. Our studies also showed that knockdown of ATP13A2 in colon cancer cells decreased the self-renewal ability of cells, and this capability was rescued by ATP13A2 overexpression. Taken together, these findings suggest that ATP13A2 is involved in colon cancer initiation. Parkinson’s disease and colon cancer share common genetic predispositions, such as *PINK1*, which is activated in opposite directions in neuronal and cancer tissues [25]. In this study, we not only identified a novel prognostic biomarker for colon cancer, but also a potential common genetic pathway between colon cancer initiation and Parkinson’s disease.

The localization of ATP13A2 within the lysosomal membrane implies that it plays a role in modulating autophagy [26]; therefore, we analyzed human colon cancer specimens. We found that low expression levels of ATP13A2 are closely related to high expression levels of LC3 and SQSTM1, both of which are degraded by lysosomal acid phosphatase following the fusion of the autophagosome with the lysosome. In cortical neurons, knockdown of ATP13A2 increased the levels of LC3 and SQSTM1 and decreased the level of cellular autophagy [27]. Therefore, we investigated the role of ATP13A2 in autophagy regulation in colon cancer cells. As expected, LC3 expression and puncta, as well as SQSTM1 expression, accumulated in ATP13A2-knockdown HCT-116 cells, indicating that autophagic flux was blocked. Other studies have shown that the loss of ATP13A2 leads to decreased mitochondrial dysfunction by blocking autophagy, which is responsible for PD pathogenesis [19]. Autophagy has emerged as a requirement for the maintenance of stemness in both normal tissue stem cells [28] and cancer stem cells [29, 30]. In line with this, our results indicate that the stemness of colon cancer cells was increased by high levels of ATP13A2, but this phenomenon was reversed by bafilomycin A1, a repressor of the formation of autolysosomes, suggesting that ATP13A2 knockdown decreased the stemness of colon cancer cells by blocking the late autophagic progress. Taken together, these results suggest that ATP13A2 contributes to colon cancer stemness through regulation of the autophagic process. We have also demonstrated that the knockdown of ATP13A2 abolished the stemness of cancer cells in vitro and that targeting ATP13A2 in colon cancer cells decreased the volume of the tumor xenograft in vivo*.* As such, we conclude that targeting ATP13A2 in colon cancer may be a good strategy for killing cancer stem cells and for inhibiting non-cancer stem cells by blocking autophagic flux.

## Conclusion

We found that Parkinson’s disease-associated gene *ATP13A2* promotes tumorigenesis through regulation of autophagy flux, which is responsible for the patient with PD was associated with lower risks of acquiring colon cancer. Additionally, ATP13A2 is not only a novel prognostic biomarker for colon cancer but also a potential target for colon cancer therapy.

## Supplementary Information

**Additional file 1: Figure S1.** The protein level of ATP13A2 was detected in normal colon epithelial cell line CCD 841CoN and human colorectal cancer cell lines, SW480 and HCT-116 by western blotting. **Table S1.** The Sequence of primers used in Parkinson’s disease gene mRNA real-time q-PCR.

## Data Availability

The datasets used and/or analyzed during the current study are available from the corresponding author on reasonable request.
