# Supplementary material for: Knockdown of Parkinson’s disease-related gene ATP13A2 reduces tumorigenesis via blocking autophagic flux in colon cancer
Source: Cell Biosci. 2020 Dec 11;10:144. doi: 10.1186/s13578-020-00506-z (PMC7731751; doi:10.1186/s13578-020-00506-z)
Supplement: Supplementary file 1 — Additional file 1: Figure S1. The protein level of ATP13A2 was detected in normal colon epithelial cell line CCD 841CoN and human colorectal cancer cell lines, SW480 and HCT-116 by western blotting. Table S1. The Sequence of primers used in Parkinson’s disease gene mRNA real-time q-PCR. [file 13578_2020_506_MOESM1_ESM.docx]

**Figure**


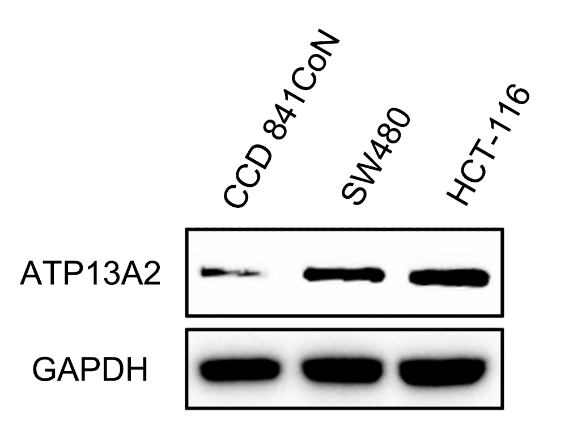


**Figure S1**

The protein level of ATP13A2 was detected in normal colon epithelial cell line CCD 841CoN and human colorectal cancer cell lines, SW480 and HCT-116 by western blotting.

**Table**

| Primer | Sequence forward（5′–3′） | Sequence Reverse （5′–3′） |
| --- | --- | --- |
| PARK1/4 SCNA | AAACCAAGGAGGGAGTGGTG | CTGTCTTCTGGGCTACTGCTG |
| PARK2 Parkin | CTGACACCAGCATCTTCCAG | CCAGTCATTCCTCAGCTCCT |
| PARK5 UCHL1 | GCCAATGTCGGGTAGATGAC | AGCGGACTTCTCCTTGCTC |
| PARK6 PINK1 | CAAGAGAGGTCCCAAGCAAC | GGCAGCACATCAGGGTAGTC |
| PARK7 DJ-1 | TGGCTAAAGGAGCAGAGGAA | ATGACCACATCACGGCTACA |
| PARK8 LRRK2 | GAGCACGCCTCCAAGTTATT | AGAAGTGACCAACCCACCTG |
| PARK9 ATP13A2 | TGGCTGGCTGACCACTACTAC | AGTCTGGCTTTGCTTTCTGG |
| PARK15 | TACCCGACAAGCACTGAACC | AAGACGGAACGAACATCCAG |
| GBA | CTTCTGCTGGGCTGTTGAGT | TACTGTTGGCGAGGGTAGGA |
| GAPDH | GGAGCGAGATCCCTCCAAAAT | GGCTGTTGTCATACTTCTCATGG |
| VIMENTIN | GCCCTAGACGAACTGGGTC | GGCTGCAACTGCCTAATGAG |
| MMP2 | TGACTTTCTTGGATCGGGTCG | AAGCACCACATCAGATGACTG |
| MMP9 | TGTACCGCTATGGTTACACTCG | GGCAGGGACAGTTGCTTCT |
| E-cadherin | CGAGAGCTACACGTTCACGG | GGGTGTCGAGGGAAAAATAGG |

**Table S1** The Sequence of primers used in Parkinson’s disease gene mRNA real-time q-PCR.
